# Survival outcome of esophagectomy and chemoradiotherapy for resectable esophageal squamous cell carcinoma in patients >75 years of age

**DOI:** 10.1111/1759-7714.15329

**Published:** 2024-06-19

**Authors:** Shuhei Mayanagi, Masazumi Inoue, Kazunori Tokizawa, Kunihiro Fushiki, Takahiro Tsushima, Tomoya Yokota, Kentaro Yamazaki, Hirofumi Yasui, Yasuhiro Tsubosa

**Affiliations:** ^1^ Division of Esophageal Surgery Shizuoka Cancer Center Hospital Shizuoka Japan; ^2^ Division of Gastrointestinal Oncology Shizuoka Cancer Center Hospital Shizuoka Japan

**Keywords:** esophageal cancer, esophagectomy, definitive chemoradiotherapy

## Abstract

**Background:**

The gold standard for resectable, locally advanced esophageal squamous cell carcinoma (ESCC) is surgery‐based treatment; however, it is unclear whether esophagectomy or chemoradiotherapy is suitable for older patients. This retrospective study aimed to identify the treatment outcomes of surgery‐based therapy versus definitive chemoradiotherapy (dCRT) as an initial treatment for older patients with resectable, locally advanced ESCC.

**Methods:**

Data from 434 patients who received radical treatment for resectable, locally advanced ESCC were collected from January 2011 to December 2020. Of the patients >75 years of age, 49 underwent radical esophagectomy and 26 received dCRT. Survival was compared between the surgery and dCRT groups.

**Results:**

The mean ages of the surgery and chemoradiotherapy groups were 77.3 and 78.8 years, respectively. Differences in overall survival (OS) between the two groups were not statistically significant (3‐year OS: surgery 66.2%, dCRT 55.7%, *p* = 0.236). Multivariate analysis for OS showed a hazard ratio of 1.229 for dCRT versus surgery (90% confidence interval 0.681–2.217). OS did not differ between the groups in any of the performance statuses. For patients who were able to receive chemotherapy using fluorouracil and cisplatin, OS tended to be better in the surgery group, but the difference was not statistically significant (3‐year OS: surgery 68.1%, dCRT 51.8%, *p* = 0.117).

**Conclusions:**

There was no clear difference in survival outcome between surgery‐based therapy and dCRT as an initial treatment for esophageal cancer in older patients. Either treatment may be an option for older patients.

## Graphical abstract

This retrospective study identified the treatment outcomes of surgery‐based therapy versus definitive chemoradiotherapy as an initial treatment for older patients with resectable, locally advanced esophageal squamous cell carcinoma. There was no clear difference in survival outcome between surgery‐based therapy and definitive chemoradiotherapy as an initial treatment for esophageal cancer in older patients.

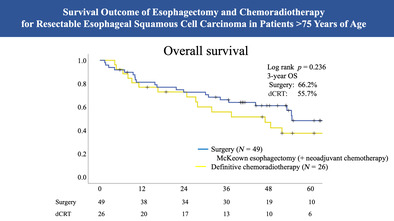

## INTRODUCTION

Surgery‐based treatment is the gold standard for resectable, locally advanced esophageal squamous cell carcinoma (ESCC). In recent years, improved survival outcomes have been reported with preoperative chemoradiotherapy^1^ or chemotherapy^2^, ^3^ followed by esophagectomy. Conversely, good survival outcomes have been reported for definitive chemoradiotherapy (dCRT) as an alternative, with reduced adverse events, in addition to advances in radiation technology and supportive care.^4^ In patients who underwent dCRT for locally advanced ESCC, 3‐year esophagectomy‐free survival and 3‐year overall survival (OS) were 63.6% and 74.2%, respectively, indicating that dCRT is comparable to surgery‐based therapy. However, because these treatment modalities, esophagectomy and dCRT, are completely different, randomized controlled trials comparing them are not feasible. Direct comparisons of survival outcomes, including post‐treatment quality of life, do not exist and no clear evidence has been presented.

Furthermore, the clinical trials were conducted in younger patients, and it is unclear whether esophagectomy or dCRT is optimal treatment, especially for older patients with resectable ESCC. In clinical practice, the standard regimen of chemotherapy, whether preoperative or in combination with radiotherapy, is often not available for older patients because of poor performance status or inadequate organ function. The incidence of treatment‐related adverse events and the risk of their severity are higher, particularly in older patients, and their management can often be a struggle. Because it may lead to a significant decline in postoperative quality of life in vulnerable older patients and trigger other morbidities, such as pneumonia, treatment choice is often difficult, even in patients with indications for surgery.

The purpose of this retrospective study was to identify the treatment outcomes of surgery‐based therapy versus dCRT in older patients with resectable, locally advanced ESCC.

## METHODS

This study was conducted in accordance with the ethical principles of the Declaration of Helsinki and was approved by the Institutional Review Board of Shizuoka Cancer Center Hospital (J2022‐209‐2022‐1‐3).

### Patients

**FIGURE 1:**
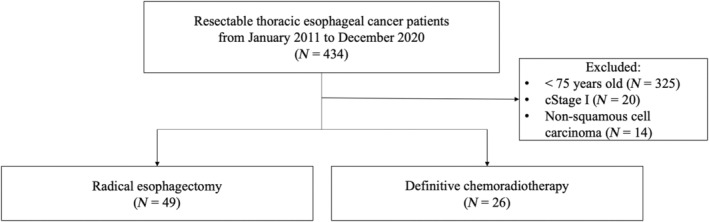
Flowchart of patient selection. In total, 75 patients who received radical treatment for locally advanced thoracic esophageal squamous cell carcinoma were included in this retrospective analysis. Data from 49 patients in the surgery group and 26 patients in the chemoradiotherapy group were analyzed, respectively.

In total, 434 consecutive patients received radical treatment for thoracic esophageal cancer at Shizuoka Cancer Center Hospital from January 2011 to December 2020 (Shizuoka, Japan) (Figure 1). Furthermore, 325 patients aged 75 years, 20 patients with cStage I, and 14 patients with adenocarcinoma were excluded from the study. Data from 75 older patients with thoracic ESCC were included in this retrospective study. All patients had resectable, locally advanced disease and were eligible for radical esophagectomy. In accordance with the principle of shared decision making, the patients were offered esophagectomy as standard treatment or dCRT as alternative treatment. During the decision‐making process in the treatment selection, a surgical oncologist and a medical oncologist explained in detail the treatment methods, complications, and post‐treatment course of the esophagectomy and dCRT, respectively. If the patient wanted organ preservation, dCRT was performed as the initial treatment. Among them, 49 patients underwent esophagectomy and 26 patients received concurrent chemoradiotherapyon request. Clinical staging was evaluated through esophagoscopy, computed tomography (CT), and (18)F‐fluorodeoxyglucose positron emission tomography before initial treatment, according to the tumor–node–metastasis classification (Amercan Joint Committee on Cancer 8th edition).

### Surgical procedure and neoadjuvant chemotherapy

For radical surgery, McKeown subtotal thoracic esophagectomy was performed with two‐ or three‐field lymph node dissection.^5^, ^6^ In the transthoracic approach, video‐assisted thoracoscopic surgery was typically performed in the left decubitus position. The abdominal approach was typically laparotomy. Standard two‐field lymph node dissection (D2) comprised removal of the mediastinal lymph nodes, including the bilateral recurrent nerve lymph nodes and abdominal lymph nodes, including the pericardial lymph nodes and lymph nodes along the lesser curvature and the left gastric artery. Three‐field lymph node dissection (D3) involved dissection of the bilateral supraclavicular lymph nodes. Reconstruction was performed using a gastric tube with layer‐to‐layer hand‐sewn cervical anastomosis through the posterior sternal route. If a gastric tube was not available, a pedicled jejunal flap was used.^7^ Because invasiveness of pedicled jejunal flap reconstruction is high for older patients, a two‐stage surgery can be performed, in which reconstruction is usually performed approximately 3 weeks after the first surgery for esophagectomy only. A feeding catheter was placed through the anterior wall of the gastric tube or jejunum.

Neoadjuvant treatment was administered for locally advanced squamous cell carcinoma, in accordance with the Japanese esophageal cancer practice guidelines.^8^ Neoadjuvant chemotherapy comprised two courses of cisplatin + 5‐fluorouracil (FU) based on the JCOG9907 trial (cisplatin 80 mg/m^2^ on days 1 and 22 + 5‐FU 800 mg/m^2^ on days 1–5 and 22–26).^9^ Upfront surgery was performed in patients with renal dysfunction or other conditions that made chemotherapy unsuitable.

### Chemoradiotherapy

As initial treatment, patients received standard concurrent chemoradiation, 50.4 Gy in 28 fractions per RTOG94‐05^4^, ^10^, ^11^, ^12^ or 60 Gy in 30 fractions per JCOG0303.^13^ The majority of concurrent chemotherapy comprised two courses of cisplatin + 5‐FU(RTOG94‐05 [cisplatin 75 mg/m^2^ on days 1 and 29 + 5‐FU 1000 mg/m^2^ on days 1–4 and 29–32]) or JCOG0303 (cisplatin 70 mg/m^2^ on days 1 and 29 + 5‐FU 700 mg/m^2^ on days 1–4 and 29–32). Four weeks after completion of radiotherapy, two additional cycles of chemotherapy (days 57 and 85) were administered. If cisplatin + 5‐FU combination therapy was not feasible, concurrent chemotherapy comprised two courses of nedaplatin + 5‐FU (nedaplatin 90 mg/m^2^ on days 1 and 29 + 5‐FU 800 mg/m^2^ on days 1–5 and 29–33) or weekly paclitaxel (paclitaxel 50 mg/m^2^ on days 1, 8, 15, 22, 29, and 36).

Gross tumor volume was defined as the volume of the primary tumor on CT scan and esophagoscopy. Metastatic lymph nodes were defined as measuring ≥1 cm on the long axis. In clinical practice, target primary tumor volume includes a 2‐cm margin craniocaudally and 0.5–1.0‐cm margin radically, considering subclinical extension. When the primary tumor is located in the upper esophagus, the clinical target volume includes the regional lymph nodes from the neck to the bifurcation of the trachea. By contrast, when the primary tumor is located in the middle or lower esophagus, the clinical target volume includes regional lymph nodes from the upper mediastinum to the left gastric artery.

The indications for salvage treatment, surgery,^6^ or endoscopic resection were considered for residual lesions in primary esophageal tumors or regional lymph nodes after completion of dCRT.

### Follow‐up

All patients who underwent surgery or received dCRT were followed up for at least 5 years. Tumor markers (carcinoembryonic antigen and squamous cell carcinoma antigen), esophagogastroscopy, and CT (cervix, chest, and abdomen) were evaluated at least every 3 months for year 1, every 4 months for year 2, and every 6 months from the years 3–5.

### Statistical analyses and outcome definitions

Baseline clinical variables are expressed as means and standard deviations for continuous variables or frequencies, and categorical variables are expressed as proportions. Statistical analyses were performed using the Statistical Package for the Social Sciences software version 27 (IBM Corporation). Clinical variables were analyzed using the *χ* ^2^ test and Fisher's exact test. *p* < 0.05 was considered statistically significant. Postoperative complications (in‐hospital complications during index admission within 30 days postoperatively) were defined as follows: arrhythmia, acute cardiac syndrome, acute heart failure, pneumonia, atelectasis, pleural effusion, chylothorax, acute respiratory distress syndrome, and anastomotic leakage. Postoperative complications were graded according to the Clavien–Dindo classification system. Early and late adverse events were evaluated according to the Common Terminology Criteria for Adverse Events version 5.0 within 90 days and after 91 days from the date of radiotherapy completion, respectively. OS was defined as the time from the date of surgery or initiation of radiotherapy until death by any cause. Post‐CRT recurrence was defined as appearance of a new local or distant lesion after confirmed complete response to dCRT. Survival curves were generated using the Kaplan–Meier survival and log‐rank tests. Since the bias in performance status for each treatment modality has a significant impact on survival, a stratified analysis was performed for each performance status. In addition, we analyzed the cases who were able to receive the same intensity of therapy as younger patients, that is, 5‐FU and cisplatin therapy. Treatment modalities and clinical factors associated with prognosis, such as performance status and clinical stage, were evaluated by multivariate analysis using the Cox proportional hazards model.

## RESULTS

Original data from 75 patients are shown in Table 1. The mean ages of the surgery and chemoradiotherapy groups were 77.3 and 78.8 years, respectively (*p* = 0.037). Performance status was poorer in the dCRT group than that in the surgery group, with performance status 1–2 in 58% of all patients in the dCRT group. In the dCRT group, the median creatinine clearance of 65.1 mL/min was better than that in the surgery group (*p* = 0.029). The dCRT group tended to have fewer patients with lower esophageal cancer and more patients with upper esophageal cancer than the surgery group. Differences in sex, Charlson comorbidity index, social background, or clinical stage were not significant. In total, 61% (30/49) of the surgery group received neoadjuvant chemotherapy, including 5‐FU and cisplatin. The dose intensities of cisplatin and 5‐FU as neoadjuvant chemotherapy were 84% and 88%, respectively.

**TABLE 1:** Characteristics of study patients.

		Surgery group		dCRT group		
		*n* = 49		*n* = 26		*p* value
Age, mean ± SD (years)		77.3 ± 2.0		78.8 ± 4.0		0.037
Sex	Male	41	84%	24	92%	0.478
	Female	8	16%	2	8%	
Performance status	0	34	69%	11	42%	0.042
	1	15	31%	14	54%	
	2	0	0%	1	4%	
Charlson Comorbidity Index	0	40	82%	20	77%	0.635
	1	1	2%	2	8%	
	2	7	14%	3	12%	
	3	0	0%	0	0%	
	4	1	2%	1	4%	
Creatinine clearance, mean ± SD (mL/min)		58.7 ± 12.2		65.1 ± 11.3		0.029
Social background	Living alone	7	14%	3	12%	1.000
Location	Ut	4	8%	8	31%	0.084
	Mt	29	59%	18	69%	
	Lt	25	51%	11	42%	
cT	1	6	12%	2	8%	0.434
	2	8	16%	2	8%	
	3	35	71%	22	85%	
cN	0	13	27%	9	35%	0.836
	1	22	45%	9	35%	
	2	12	24%	7	27%	
	3	2	4%	1	4%	
cM	0	48	98%	26	100%	1.000
	1	1	2%	0	0%	
cStage	II	17	35%	10	38%	0.803
	III	32	65%	16	62%	
Neoadjuvant therapy	(−)	19	39%			
	CF	29	59%			
	NF	1	2%			
Radiation, mean ± SD (Gy)				56.5 ± 4.9		
Concurrent chemotherapy	CF			19	73%	
	PTX			4	15%	
	NF			3	12%	

Abbreviations: CF, cisplatin + 5‐fluorouracil; cM, clinical distant organ and lymph node metastasis; cN, clinical grading of lymph node metastasis; cStage, clinical stage; cT, clinical depth of tumor invasion; dCRT, definitive chemoradiotherapy; NF, nedaplatin + 5‐fluorouracil; PTX, paclitaxel.

All patients in the surgery group underwent McKeown esophagectomy and 67% (33/49) underwent video‐assisted thoracoscopic surgery (Table 2). Lymph node dissection was omitted in 22% (11/49) of cases, with lymph node dissection less than D2. In total, 14% (7/49) of cases were reconstructed with the jejunum because gastric tubes were not available. In the seven cases of incomplete resection, a microscopic residual tumor (R1) was found in five cases and a macroscopic residual tumor (R2), in which the primary tumor invaded the aorta, was found in two cases. One patient with R2 resection underwent postoperative dCRT with cisplatin + 5‐FU for residual tumor, and the other patient with R2 resection received systemic chemotherapy with nivolumab. Although overall the postoperative complications rate ≥Clavien–Dindo grade III was 51% (25/49), including pneumonia (*n* = 6), pleural effusion (*n* = 3), surgical site infection (*n* = 2), and anastomotic stricture (*n* = 14), 30‐day mortality after esophagectomy was 0/49 (0%).

**TABLE 2:** Surgical outcomes.

		Surgery group	
		*n* = 49	
Esophagectomy	VATS	33	67%
	Open	16	33%
Lymph node dissection	D1	11	22%
	D2	20	41%
	D3	18	37%
Two‐stage operation	One‐stage	42	86%
	Two‐stage	7	14%
Reconstruction	Gastric tube	42	86%
	Pedicled jejunum	7	14%
Operative time, mean ± SD (min)		392 ± 72	
Volume of blood loss, mean ± SD (mL)		254 ± 207	
R0 resection	R0	42	86%
	R1/2	7	14%
Postoperative complication	Pneumonia (CD ≧ Gr3)	6	12%
	Pleural effusion (CD ≧ Gr3)	3	6%
	RLNP (CD ≧ Gr1)	8	16%
	Anastomotic leakage (CD ≧ Gr2)	6	12%
	Anastomotic stricture (CD ≧ Gr3)	14	29%
	Chylothorax (CD ≧ Gr2)	5	10%
	Surgical site infection (CD ≧ Gr3)	2	4%
Operative mortality		0	0%
Hospital stay, mean ± SD (days)		26.8 ± 19.4	

Abbreviations: CD, Clavien–Dindo; D: extent of lymph node dissection; Gr: grade; R0: no residual tumor, R1/2: microscopic or macroscopic residual tumor; RLNP, recurrent laryngeal nerve palsy; VATS, video‐assisted thoracoscopic surgery.

In the dCRT group, concurrent chemotherapy comprised cisplatin and 5‐FU in 73% (19/26), paclitaxel in 15% (4/26), and nedaplatin and 5‐FU in 12% (3/26) of cases (Table 1).The dose intensities of cisplatin and 5‐FU as concurrent chemotherapy were 86% and 91%, respectively. The mean radiation dose was 56.5 Gy and radiation dose intensity for each concurrent chemotherapy regimen was 94% for cisplatin and 5‐FU, 100% for paclitaxel, and 100% for nedaplatin and 5‐FU. Only one patient discontinued radiotherapy at 46.8/50.4 Gy because of esophagitis, appetite loss, and thrombocytopenia. Grade 4 leukopenia and grade 4 hyponatremia were adverse events in 19% (5/26) and 8% (2/26) of cases, respectively (Table 3). Although one patient each had heart failure and pneumonitis as late radiation‐related toxicity in the dCRT group, there was no treatment‐related death. The completion rate of radiotherapy was 96% (25/26). The complete response rate based on the RECIST criteria was 65% (17/26). In total, five patients received additional chemotherapy after dCRT, including cisplatin + 5‐FU (*n* = 4) and nedaplatin + 5‐FU (*n* = 1). No patient underwent salvage surgery for local residual or recurrence disease after dCRT due to unresectable lesions that invaded the trachea, poor general condition after dCRT, or patient refusal of salvage surgery.

**TABLE 3:** Adverse events.

dCRT group	CTCAE grade			
*n* = 29	2	3	4	5
White blood cell decreased	2	7	5	0
Anemia	3	1	0	0
Platelet count decreased	1	6	0	0
Hyponatremia	0	1	2	0
Acute kidney injury	1	1	0	0
Febrile neutropenia	‐	4	0	0
Appetite loss	5	4	0	0
Nausea	2	0	‐	‐
Esophagitis	7	4	0	0
Fatigue	3	0	0	‐
Diarrhea	1	1	0	0
Constipation	1	0	0	0
Pneumonitis	3	1	0	0
Dermatitis	1	0	0	0
Mucositis oral	1	2	0	0

Abbreviations: CTCAE, common terminology criteria for adverse events; dCRT, definitive chemoradiotherapy.

Post‐treatment recurrence or disease progression was observed in 20 (41%) and 17 (65%) patients in the surgery and dCRT groups, respectively. Of the 20 recurrences in the surgery group, five were only regional lymph nodes and 15 were distant organs, including the lung, liver, bone, para‐aortic lymph nodes, and pleural dissemination. By contrast, 50% (8/16) relapses occurred in patients in the dCRT group who achieved complete response after initial treatment; only one of these relapse lesions was a primary esophageal tumor regrowth, and all of the remaining relapse lesions were distant metastases of the lung, liver, para‐aortic, and hilar lymph nodes.

**FIGURE 2:**
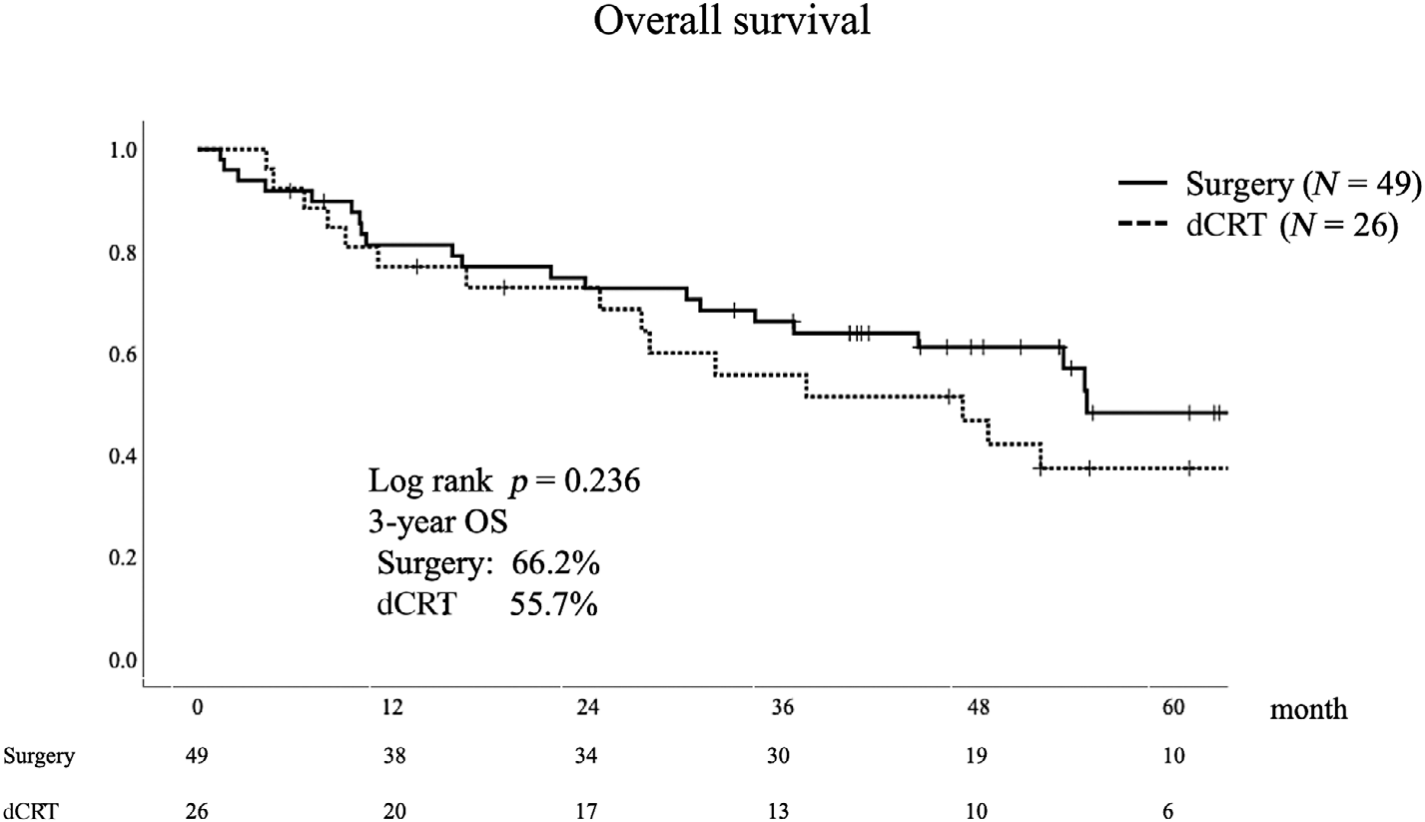
Kaplan–Meier graph of overall survival. The 3‐year overall survival (OS) rates after surgery and definitive chemoradiotherapy (dCRT) were 66.2% and 55.7%, respectively.

**FIGURE 3:**
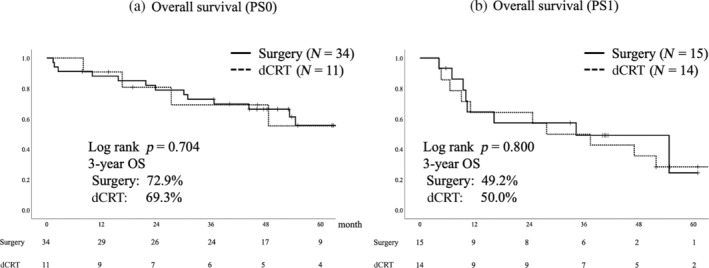
Kaplan–Meier graph of overall survival by performance status. Overall survival did not differ between groups in patients with either (a) performance status 0 (PS0) or (b) performance status 1 (PS1). dCRT, definitive chemoradiotherapy; OS, overall survival.

**FIGURE 4:**
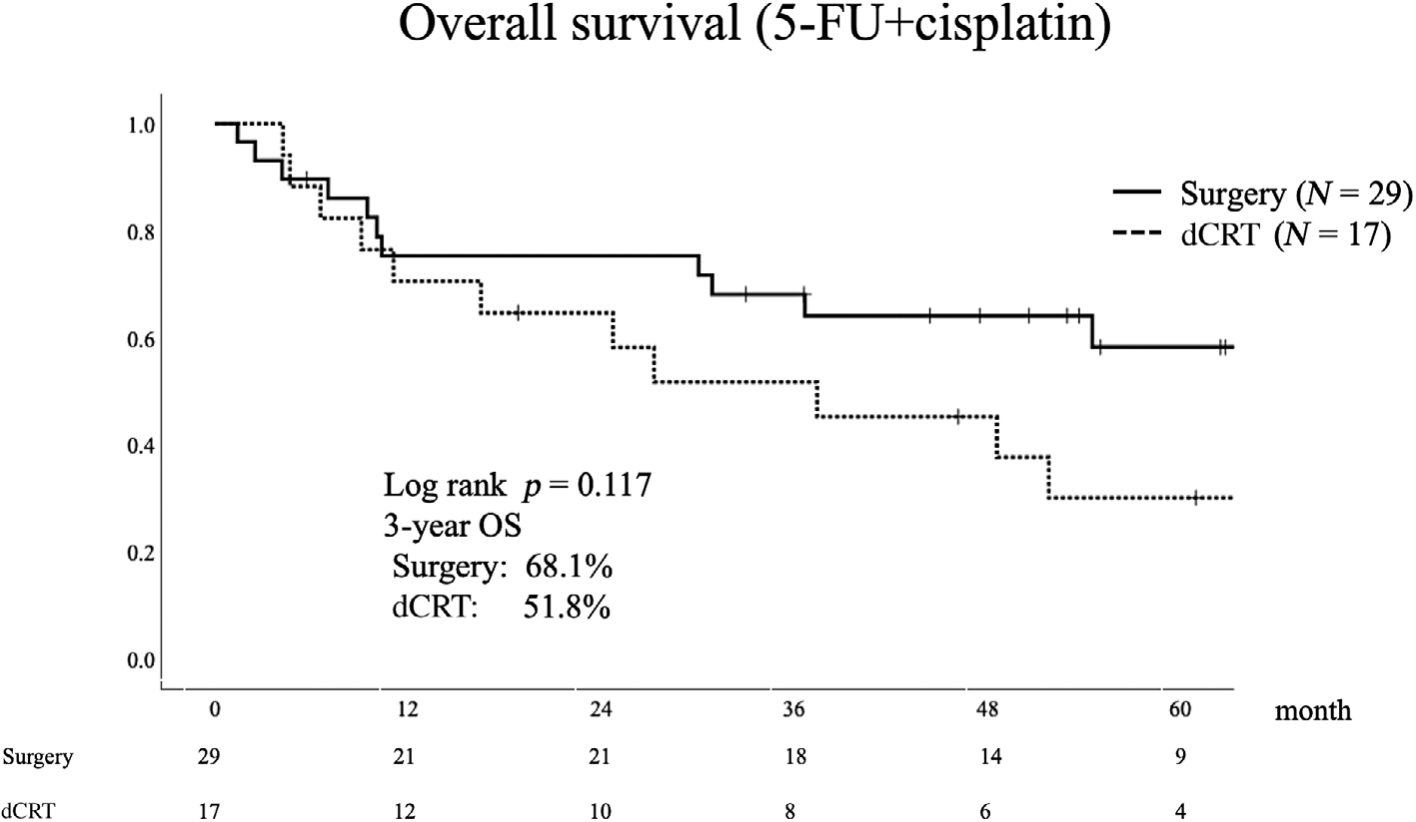
Kaplan–Meier graph of overall survival in the cisplatin + 5‐FU group. There was a trend toward better overall survival in the surgery group in patients receiving 5‐FU and cisplatin therapy. 5‐FU, 5‐fluorouracil; dCRT, definitive chemoradiotherapy; OS, overall survival.

Differences in OS between the two groups were not statistically significant (3‐year OS: surgery 66.2%, dCRT 55.7%, *p* = 0.236, median follow‐up period 38.3 months) (Figure 2). Multivariate analysis for OS showed a hazard ratio of 1.229 for dCRT versus surgery (90% confidence interval 0.681–2.217) (Table 4). In the surgery and dCRT groups, 22% (11/49) and 50% (13/26) of deaths were due to esophageal cancer, respectively. In the surgery group, six patients (12%) died for reasons other than esophageal cancer: four from pneumonia and one each from cerebral stroke, lung cancer, and old age. By contrast, the dCRT group had four deaths in 15% of the patients due to myocardial infarction, pneumonia, or gastric cancer. In the stratified analysis by performance status, OS did not differ between the groups in any of the performance statuses (Figure 3). For patients who were able to receive the same intensity of treatment as younger patients, that is, 5‐FU and cisplatin therapy, OS tended to be better in the surgery group; however, the difference was not statistically significant (3‐year OS: surgery 68.1%, dCRT 51.8%, *p* = 0.117) (Figure 4).

**TABLE 4:** Multivariate analysis for overall survival.

		Hazard	*p* value	90% CI	
Modality	dCRT	1.229	0.566	0.681	2.217
	Surgery	1.000			
PS	1	1.992	0.052	1.110	3.574
	0	1.000			
cStage	3	1.605	0.206	0.868	2.969
	2	1.000			

Abbreviations: CI, confidence interval; cStage, clinical stage; dCRT, definitive chemoradiotherapy; PS, performance status.

## DISCUSSION

In an aging society, the treatment of older patients with cancer is one of the most challenging issues. Because older patients cannot be treated with the same intensity as younger patients, it is often difficult to treat them based on evidence from clinical trials. This retrospective study showed no significant difference in survival outcomes between surgery‐based therapy and dCRT, and both treatments could be considered treatment options for older patients. In practice, in addition to treatment outcomes and adverse events for each modality, consideration of patients' social backgrounds, views on life and death, and ethical considerations may be necessary. For treating esophageal cancer in older patients, treatment choices should be made on an individual basis from a comprehensive perspective.

An analysis of the National Database of Hospital‐based Cancer Registries in Japan, a rapidly aging population, reported survival outcomes with surgery for older patients with esophageal cancer.^14^ Compared to dCRT, esophagectomy improved survival in patients with cStage II–III esophageal cancer (age 75–79 years); however, there was no superiority of surgery in patients >80 years of age. Although this report is a large dataset, it does not provide information on the performance status and comorbidity of each patient, and there was significant selection bias because the physical condition could not be evaluated. In our study, we compared survival outcomes by stratification analysis and multivariate analysis to eliminate selection bias. The difference in survival benefit between surgery and dCRT may not be significant in older ESCC patients with the same physical condition. On the other hand, surgery‐based therapy might be more conducive to survival in older patients who could receive doublet chemotherapy. A more detailed study with a large cohort is needed, focusing on older patients who are eligible for the intense chemotherapy.

Minimal invasive surgery or the enhanced recovery after surgery concept has become popular in recent years for esophagectomy. Minimally invasive surgery and refined perioperative management may reduce complications and expand the indications for surgery in older patients, who were considered difficult to treat. Surgical invasiveness may be further reduced by omitting prophylactic lymph node dissection or two‐stage surgery. Postoperative complications have an increasingly negative impact on long‐term prognosis in older patients,^15^ and patients with postoperative complications tended to have poorer survival outcomes in this study (data not shown), therefore avoiding postoperative complications, particularly in older patients, is important.

Chemotherapy may be used in combination with surgery‐based treatment or radiation therapy for treating ESCC and may be difficult to administer to older patients. Preoperative adjuvant therapy followed by surgery is the standard of care; however, doublet or triplet chemotherapy may not be indicated in older patients. The reported rates of neoadjuvant therapy in combination with preoperative adjuvant chemotherapy or radiation in patients >75 years of age ranged from 38% to 69%,^16^, ^17^, ^18^, ^19^ and the 60% rate obtained in this study is comparable. By contrast, the dCRT group in this study had a manageable frequency and severity of adverse events with no treatment‐related deaths; however, electrolyte disorders, such as hyponatremia, may require attention in older patients. Although the percentage of complete response after dCRT is not low at 65%, dCRT did not provide satisfactory long‐term results in older patients compared with younger patients. Treatment regimens focusing on older patients have not been established for either preoperative adjuvant therapy or dCRT, and needs to be resolved in the future. 5‐FU, leucovorin and oxaliplatin may be a potential combination regimen with radiotherapy indicated for older patients.^20^ The results of clinical trials of dCRT in older patients showed the superiority of radiation therapy with S‐1 over radiation alone.^21^ Treatment development is needed in patients who are ineligible for dCRT with platinum and FU or taxane.

For patients with residual or recurrent cancer after dCRT, salvage surgery is the only remaining treatment option. In the JCOG0909 trial,^4^ a single‐arm confirmatory study on dCRT, including salvage treatment, conducted in Japan, the rate of salvage surgery for resectable ESCC was 29%, whereas no salvage surgery was performed in our study of older patients. In this study, only two (8%) patients had recurrence limited to the primary tumor or regional lymph node that could have been treated with salvage surgery. These patients were not operated on because of patient refusal or poor general condition after dCRT. Although salvage surgery has become relatively safe because of the omission of lymph node dissection,^6^ it generally carries a high risk of postoperative complications. In addition, older patients have often decreased organ function and performance status associated with dCRT, which may limit the indications for salvage surgery.

This study had some limitations. This was a single‐center, retrospective study conducted in Asia. The chemotherapy regimens were not uniform, and neoadjuvant chemotherapy was not used in some cases in surgery‐based treatment. In addition, dCRT was not used as perioperative treatment. Treatment outcome was survival only, and the post‐treatment quality of life was not evaluated.

In conclusion, differences in survival outcome between surgery‐based therapy and dCRT as initial treatment for esophageal cancer in older patients were unclear. Either treatment may be an option for older patients.

## AUTHOR CONTRIBUTIONS

All authors had full access to the data in the study and take responsibility for the integrity of the data and the accuracy of the data analysis. Conceptualization: S.M. Methodology: S.M. Investigation: M.I, K.T., K.F, T.T., T.Y., and Y.K. Formal analysis: S.M. Project administration: Y.T. Writing–original draft: S.M. Writing–review & editing: M.I., T.T., and Y.T.

## FUNDING INFORMATION

The authors did not receive support from any organization for the submitted work.

## CONFLICT OF INTEREST STATEMENT

The authors declare no conflict of interest.

## PATIENT CONSENT STATEMENT

Informed consent was obtained from all participants.

## Data Availability

The data are not publicly available due to their containing information that could compromise the privacy of research participants.
